# A dual-modality machine learning precision diagnostic model integrated radiomics and proteomics for breast cancer

**DOI:** 10.3389/fimmu.2025.1665459

**Published:** 2025-11-06

**Authors:** Pengping Li, Yuan Liu, Ren Liu, Yuqin Huang, Ke Sun, Kexin Yin, Jiajia Lu, Lanqing Li, Shuirong Zhang, Claire Y. Tong, Jiayi Liu, Junli Gao, Zhenyu Wang

**Affiliations:** 1 The First People’s Hospital of Xiaoshan District, Xiaoshan Affiliated Hospital of Wenzhou Medical University, Hangzhou, China; 2 Zhejiang Chinese Medical University, Hangzhou, China; 3 Hangzhou Cosmos Wisdom Mass Spectrometry Center of Zhejiang University Medical School, Hangzhou, China; 4 Phillips Academy Andover, Boston, MA, United States; 5 Salisbury School, Salisbury, MD, United States

**Keywords:** breast cancer, proteomics, radiomics, machine learning, diagnosis model

## Abstract

**Background:**

This study aims to construct a dual-modal machine learning model that integrates ultrasound radiomics and plasma proteomics for the precise diagnosis of breast cancer.

**Methods:**

Using a multi-source data integration strategy, 10 protein markers and 14 ultrasound radiomics features were screened from the TCGA, CPTAC databases, and the clinical cohort (including 60 healthy controls, 60 cases of benign breast diseases, and 60 cases of breast cancer) based on plasma protein mass spectrometry and ultrasound data. A dual-modal diagnostic model was constructed in combination with machine learning algorithms.

**Results:**

The results showed that the protein marker detection model performed outstandingly in the primary screening of healthy people and breast diseases (with the highest AUC of 0.974). Still, its diagnostic performance was limited in differentiating benign and malignant diseases (AUC<0.8 under multiple algorithms). The bimodal model demonstrated excellent performance (AUC = 0.938) in differentiating benign and malignant lesions, significantly outperforming the single proteomics model (AUC = 0.830) and the radiomics model (AUC = 0.841).

**Conclusion:**

This study confirmed for the synergistic diagnostic value of plasma proteins and ultrasound images, providing a new strategy with both accuracy and accessibility for stratified diagnosis of breast cancer.

## 1 Introduction

Breast cancer is one of the most common malignant tumors in women. According to the 2024 China Cancer Report, the incidence rate (51.7%) of breast cancer is second only to lung cancer among female cancers, and its mortality rate (10.86%) ranks fifth (1). Although significant progress has been made in treatment, precision detection remains a key factor in improving survival rates. The complexity of breast cancer necessitates the identification of novel biomarkers and strategies for improved diagnosis, prognosis, and therapeutic response prediction.

At present, the commonly used clinical diagnostic methods for breast cancer, such as mammography, ultrasonography, magnetic resonance imaging, and other imaging methods, as well as histopathological examination, all have certain limitations. Although imaging examinations can detect morphological changes in the breast, they have a relatively high false positive rate, which easily leads to unnecessary further examinations. Although histopathological examination is the “gold standard” for diagnosis, it is an invasive operation that will cause certain pain to patients and is not suitable for large-scale screening. Therefore, there is a clinical need to develop noninvasive diagnostic techniques and methods that are objective, accurate, and highly sensitive to effectively improve the performance of discriminating early breast cancer lesions and providing auxiliary diagnosis.

Blood biomarkers are increasingly applied in clinical practice due to their easy acquisition, non-invasiveness, and low cost. By detecting common serum markers, they can be used for the auxiliary diagnosis, early screening, and prognosis monitoring of cancer (2). The common serum tumor markers widely used in clinical breast cancer detection include carcinoembryonic antigen (CEA), carbohydrate antigen 125 (CA125), carbohydrate antigen 153 (CA153) (3). However, many clinical trials have shown that these conventional tumor markers have insufficient specificity, limited diagnostic performance between benign breast diseases and malignant tumors, and there are certain false positives or false negatives in the diagnosis of breast cancer (4). With the rapid development of proteomics technology, tumor protein markers with specific expression have been continuously discovered (4, 5). By analyzing and comparing the proteomics of cancer patients and healthy controls, comparing the specific proteins or peptide segments that are up-regulated or down-regulated in the plasma proteome profiles of breast cancer patients, screening out differentially expressed proteins, and exploring potential new biomarkers (6). Proteomics is an effective method for mining biomarkers and can provide new strategies and targets for the discovery of breast cancer biomarkers.

On the other hand, imaging omics has emerged as a powerful tool in the field of medical imaging. By extracting and analyzing a large number of quantitative features from medical images, imaging omics can provide detailed information about the tumor’s characteristics, which is helpful for the diagnosis and prognosis of diseases. Combining imaging omics features with plasma protein markers may provide a more comprehensive understanding of breast cancer and improve the accuracy of early diagnosis.

**Figure 1:**
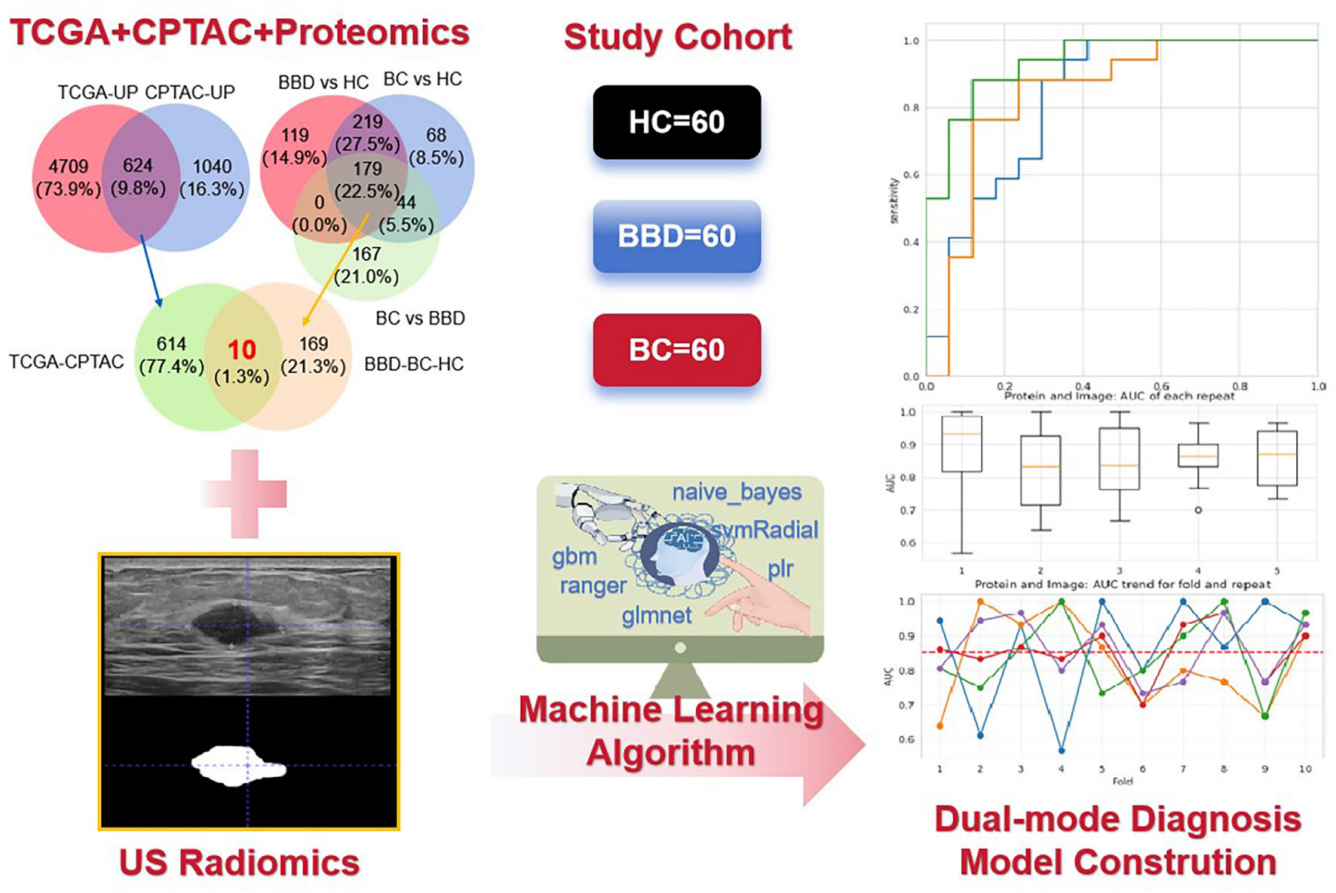
The flowchart of the key steps from data collection to model construction and performance evaluation. It illustrates the integration of ultrasound (US) radiomics from breast images and plasma proteomics data (The intersection was taken from TCGA + CPTAC + Proteomics) based on a study cohort (60 healthy controls [HC], 60 benign breast disease [BBD], 60 breast cancer [BC] patients), and the application of machine learning algorithms to construct a dual-mode diagnostic model.

Current diagnostic models for breast cancer rely on single-modal data such as imaging or proteomics, which lack robustness. This study develops a dual-modal model combining plasma proteins (with a focus on immune-related biomarkers) and ultrasound radiomics to improve diagnostic accuracy (**Figure 1**). Notably, this approach aligns with immunology research priorities by linking peripheral molecular signatures to tumor microenvironment (TME) immune dynamics. We hope that this model can improve the accuracy and efficiency of breast cancer diagnosis and provide a new approach and strategy for the clinical diagnosis of breast cancer.

## 2 Materials and methods

### 2.1 Study cohort

A total of 180 participants were enrolled, including 60 healthy controls (HC), 60 patients with benign breast disease (BBD), and 60 breast cancer (BC) patients. The details of the subjects were shown in **Supplementary Table S1**. All subjects were recruited from Hangzhou Xiaoshan District First People’s Hospital (from June 2022 to October 2023). Inclusion criteria for HC were no history of breast-related diseases and normal breast imaging and laboratory examinations. BBD patients were diagnosed via clinical, imaging, and pathological examinations (Fibroadenoma, Breast hyperplasia, Adenosis of the breast, Intraductal papilloma and other benign lesions). BC patients were pathologically confirmed as having invasive breast cancer. Exclusion criteria included a history of other malignant tumors, severe systemic diseases, or incomplete clinical data. This study has been approved by the Medical Ethics Committee of the First People’s Hospital of Hangzhou Xiaoshan District (NO. 2022-026), and all data containing patient identity information have been de-labeled.

### 2.2 Plasma protein samples

Fasting venous blood (5 mL) was collected from each participant into EDTA-coated tubes. Blood samples were centrifuged at 3000 rpm for 10 min at 4°C to separate plasma. The supernatant was carefully aliquoted and stored at -80°C until proteomic analysis. Plasma proteins were extracted using a commercial protein extraction kit optimized for plasma samples. The protein concentration was quantified by the BCA method. For digestion, 100 μg of protein was reduced with 5 mM dithiothreitol at 56°C for 30 min, alkylated with 11 mM iodoacetamide in the dark at room temperature for 15 min, and then digested with trypsin (1:50 enzyme-to-protein ratio) at 37°C overnight.

### 2.3 Proteomic analysis

The digested peptides were analyzed by Orbitrap Astral mass spectrometer (MS) (Thermo Fisher Scientific) with a C18 column (2.1×150 mm, 1.9 μm). The mass spectrometer was operated in data-independent acquisition (DIA) mode. DIA scan range: 350–1200 m/z; isolation window: 12 m/z (stepped by 1 m/z); resolution: 120,000 (full scan) and 30,000 (MS/MS); max injection time: 50 ms (full scan) and 30 ms (MS/MS). A self-built spectral library (based on BC plasma samples from our cohort) was used. Protein identification/quantification was performed via Spectronaut 16.0 (Biognosys), with the following settings: false discovery rate (FDR) < 1% at both peptide and protein levels; normalization method: total ion current.

### 2.4 Screening for protein biomarkers

Differentially expressed proteins (DEPs) were identified with an adjusted *P*-value<0.05 (padj, FDR-corrected via Benjamini-Hochberg method) and absolute fold-change (abs(FC)) > 1.5. To screen candidate protein biomarkers, we integrated DEPs from The Cancer Genome Atlas (TCGA), Clinical Proteomic Tumor Analysis Consortium (CPTAC) breast cancer datasets, and our proteomics data, taking the intersection of up-regulated proteins across these datasets.

To ensure the plasma detectability of candidate protein biomarkers, we validated each protein using annotations from authoritative databases (UniProt: https://www.uniprot.org/; TMHMM: https://services.healthtech.dtu.dk/service.php?TMHMM-2.0; THPA: https://www.proteinatlas.org/) and our in-house DIA-MS data. Proteins were categorized based on subcellular location predictions/supportive evidence (**Supplementary Table S2**). Directly secreted protein or those with plasma detection evidence were prioritized, while TMHMM-predicted extracellular proteins were rationalized based on biological secretion potential in cancer contexts. All candidates showed differential expression in our plasma cohort, confirming their plasma presence.

### 2.5 Radiomic feature extraction

Ultrasound images of breast lesions were retrospectively collected from the hospital’s medical imaging system. Radiomics features were extracted according to the International Image Biomarker Standardisation Initiative (IBSI) standards (7). Two experienced radiologists independently performed manual segmentation of the region of interest (ROI) on the images. In cases of segmentation discrepancies, a consensus was reached through joint review, and the finalized ROI was used for radiomic feature extraction. Using the segmented ROI from ultrasound images, radiomic features were extracted with ITK-SNAP 4.0 software. A total of 93 features were initially extracted, including first-order statistics, shape features, and texture features.

To reduce dimensionality and select the most relevant features, we first calculated the correlation coefficient between features. Features with a correlation coefficient > 0.8 were considered highly correlated, and one of them was removed. Then, recursive feature elimination (RFE) combined with a support vector machine (SVM) was used to further select features, with the number of features selected optimized based on the mean test accuracy.

### 2.6 Machine learning model construction

We used multiple machine learning algorithms. Protein-based model: including Gradient-Boosting Machine (GBM), Generalized Linear Models with Elastic-Net Regularization (GLMNET), Partial Least Squares Regression (PLR), Support Vector Machine with Radial Kernel (SVR), Naive Bayes (NB), and Random Forest (RF). And radiomics-based model: DecisionTreeclassifier (DT), SVM, Stochastic Gradient Descent (SGD), K-Nearest Neighbor (KNN), NearestCentroid (NC), GaussianProcess (GP), GaussianNB (GNB), AdaBoost (ABC), GradientBoosting (GBC), Xtreme Gradient Boosting (XGB).

All classifiers were included in scikit-learn (v1.6.1) and used default parameters. During the data preprocessing stage, StandardScaler transformer was used. In model select process, the full dataset was split to train (0.7) and test (0.3) dataset. The same train dataset and test dataset was inputted to all classifiers. Then 10-fold cross-validation was also performed to evaluate mean accuracy of all classifiers for the full dataset. Based on area under the receiver operating characteristic curve (AUC) and CV mean accuracy, SVM was selected for next combined analysis. The thresholds for all models were selected using the Youden Index (J=Sensitivity+Specificity-1). For the dual-modal model, we combined the selected protein biomarkers and radiomic features, and the feature combination was input into the machine learning algorithms for model training and validation. We employed SHapley Additive exPlanations (SHAP) analysis, a method rooted in cooperative game theory, to quantify the contribution of each feature (10 plasma proteins and 14 radiomic features) to the dual-modal model’s diagnostic decisions, thereby enhancing model interpretability.

### 2.7 Statistical analysis

Statistical analyses were performed using SPSS 26. Differences in protein expression levels between groups were analyzed using the Mann-Whitney U test. Raw *P*-values from this test were further adjusted using the Benjamini-Hochberg method to control the FDR, with an adjusted *P*-value (padj) < 0.05 considered statistically significant. Correlation analysis between protein biomarkers was conducted using Pearson correlation. For machine learning model evaluation, the mean and standard deviation of AUC values across 10-fold cross-validation were calculated. *P*-values < 0.05 were considered statistically significant.

## 3 Results

### 3.1 Identification of candidate protein biomarkers

**Figure 2:**
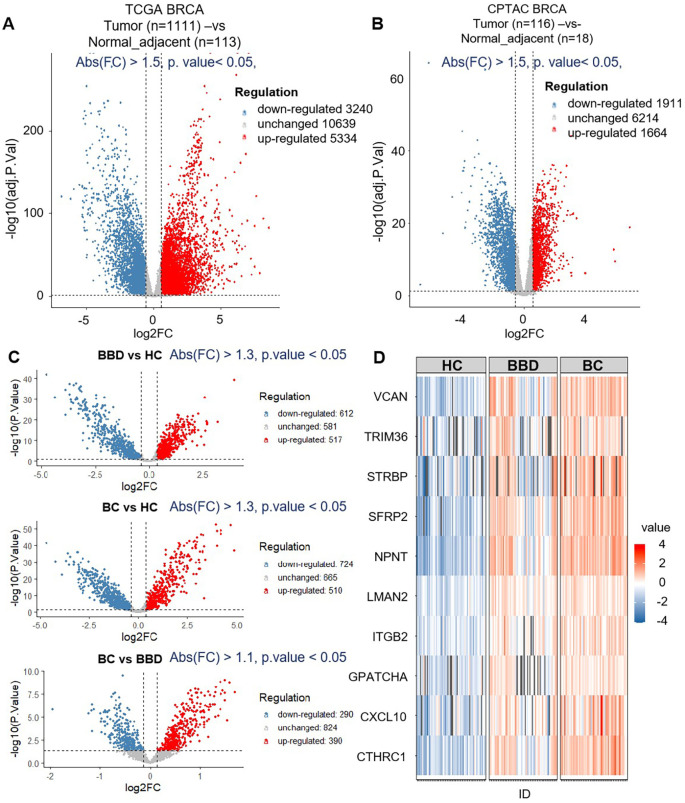
The results of biomarker discovery from the dataset (TCGA+CPTAC) and proteomics. **(A)** The volcano plot of breast tumor (n=1111) and normal adjacent (n=113) in TCGA. **(B)** The volcano plot of breast tumor (n=116) and normal adjacent (n=18) in CPTAC. **(C)** The volcano plot of differential expression proteins of healthy control (HC, n=60), benign breast disease (BBD, n=60) and breast cancer (BC, n=60) by data-independent acquisition (DIA) proteomics. **(D)** Venn diagram of the number of differentially expressed genes and differentially expressed proteins between TCGA, CPTAC and proteomics data.

To screen potential diagnostic targets, we integrated multi-source common data including TCGA and CPTAC databases, and proteomics data from our study cohort (60 HC, 60 BBD, 60 BC). As shown in **Figures 2A, B**, in the TCGA and CPTAC databases, we first screened for genes/proteins that were up-regulated in tumor tissues compared to adjacent normal tissues. The filtering thresholds were set as adjusted P-value (padj) < 0.05 and absolute fold-change (abs(FC)) > 1.5. A large number of genes showed differential expression. Among them, 3334 genes were up-regulated in tumor tissues meeting the criteria. 1664 proteins were up-regulated in tumor tissues. By taking the intersection of these up-regulated proteins from the two databases, we obtained 624 proteins that were consistently up-regulated in tumor tissues relative to adjacent normal tissues in both TCGA and CPTAC.

Then, these proteins served as candidate biomarkers for subsequent proteomics analysis based on the clinical cohort (60 HC, 60 BBD, 60 BC), as shown in **Figure 2C**. 517 proteins were up-regulated in BBD (BBD vs HC), 510 proteins were up-regulated in BC (BC vs HC), and 390 proteins were up-regulated in BC (BC vs BBD). By extracting the intersection of these up-regulated proteins from the three cohorts, we obtained 179 proteins which were then intersected with the 624 proteins mined in the previous public database, and finally obtained 10 protein markers (CTHRC1, CXCL10, GPATCH4, ITGB2, LMAN2, NPNT, SFRP2, STRBP, TRIM36, VCAN), as shown in **Figure 2D** and **Supplementary Table S2**.

**Figure 3:**
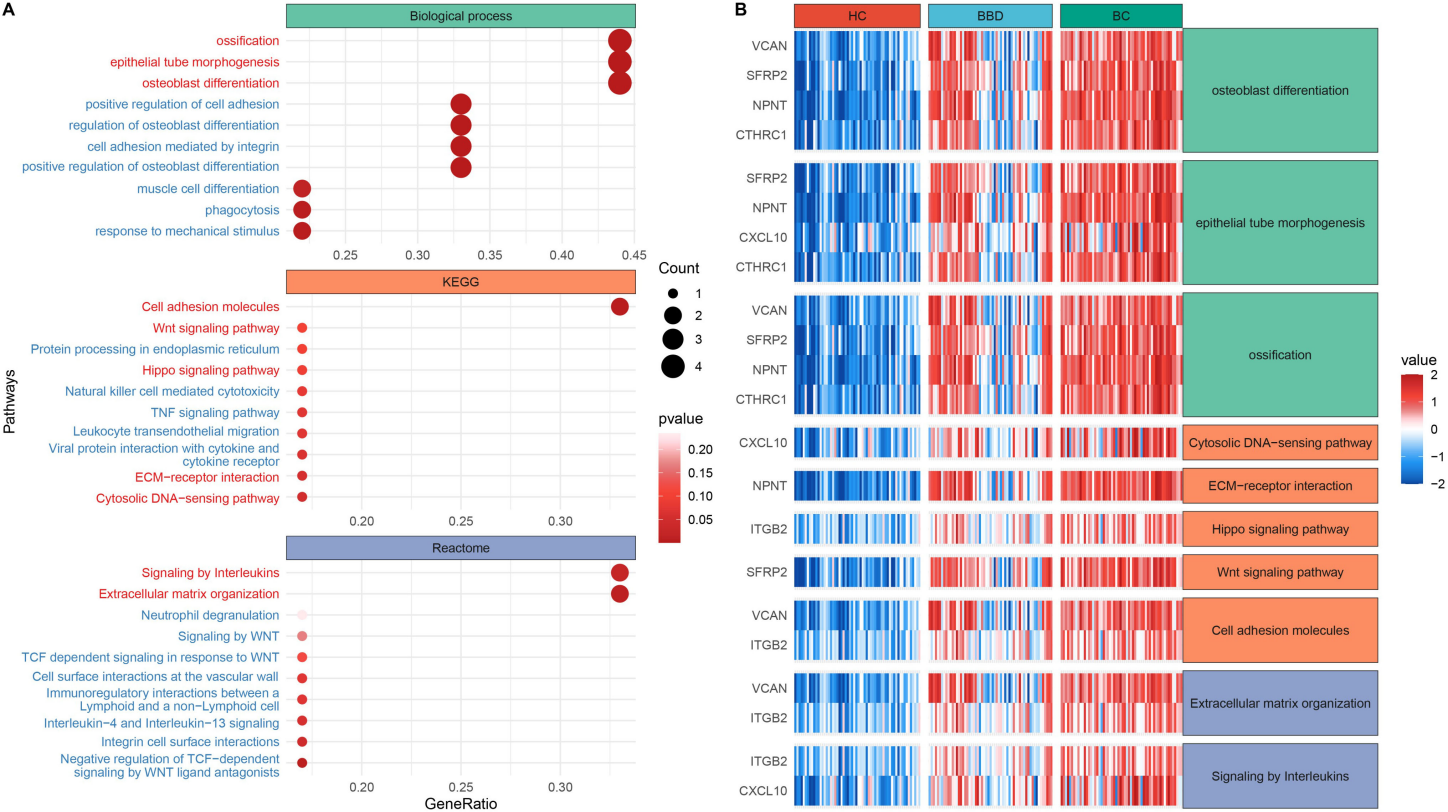
Functional enrichment analysis of candidate biomarkers. **(A)** Dot plot of enriched biological processes and pathways. **(B)** Expression heatmap across clinical groups.

Functional enrichment analysis of 10 candidate biomarkers was performed, as shown in **Figure 3**. These biomarkers were significantly enriched in processes like osteoblast differentiation, epithelial tube morphogenesis and ossification, as well as Kyoto Encyclopedia of Genes and Genomes (KEGG) pathways including Cell adhesion molecules, Cytosolic DNA-sensing pathway, extracellular matrix (ECM)-receptor interaction, Hippo signaling pathway and Wnt signaling pathway, and also reactome pathways including Extracellular matrix organization and Signaling by Interleukins. The distinct expression patterns and major enriched pathways of these 10 candidate biomarkers in different groups were shown in **Figure 3B**, laying a solid foundation for subsequent diagnostic model construction. This multi-data-source integration strategy effectively narrowed down the range of potential diagnostic targets.

### 3.2 Diagnostic performance of individual protein biomarkers

**Figure 4:**
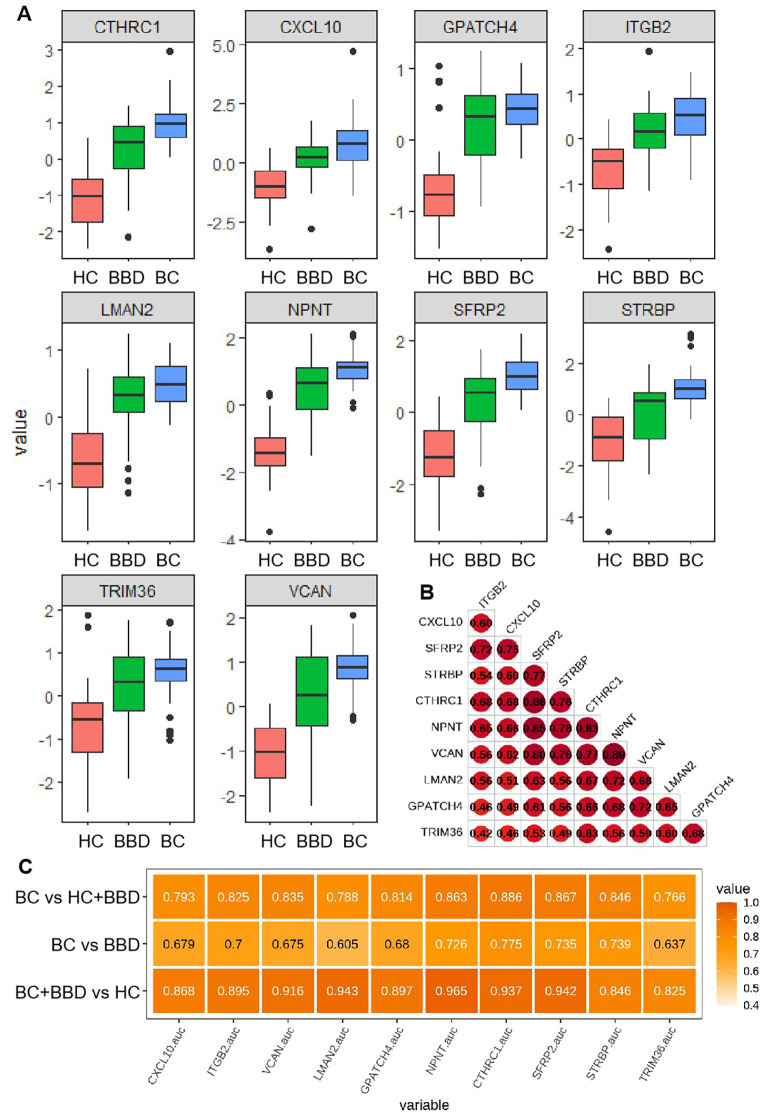
**(A)** Distribution of plasma expression levels for 10 candidate biomarkers across three groups (HC, BBD, BC). **(B)** Expression correlation matrix among the candidate biomarkers. **(C)** Performance comparison of individual candidate biomarkers in distinguishing different cohorts.

To characterize the diagnostic potential of 10 candidate protein biomarkers in breast diseases, we analyzed their expression patterns, inter-relationships, and performance in clinical cohort plasma, as shown in **Figure 4**. The 10 biomarkers showed distinct expression trends across groups, with their levels generally increasing progressively from HC to BBD and further to BC, indicating their potential to distinguish malignant from benign or healthy states, as shown in **Figure 4A**.

A correlation heatmap revealed strong positive associations among most biomarkers, such as notable correlations between CXCL10 and ITGB2, as well as CTHRC1 and NPNT, suggesting these proteins may participate in coordinated molecular pathways during breast disease development and imply shared regulatory mechanisms or functional synergies in disease progression (**Figure 4B**).

The diagnostic performance of each biomarker in different pairwise comparisons (HC + BBD vs BC, BBD vs BC, HC vs BBD + BC) was presented in **Figure 4C**. In the comparison between BC vs HC+BBD, the top 3 markers with the highest AUC are CTHRC1 (0.886), SFRP2 (0.867), and NPNT (0.863), all with AUC values over 0.85. In the comparison between BC vs BBD, the top 3 markers with the highest AUC are CTHRC1 (0.775), STRBP (0.739), and SFRP2 (0.735). In the comparison between BC+BBD vs HC, the top 3 markers with the highest AUC are NPNT (0.965), LMAN2 (0.943), and SFRP2 (0.942). It’s demonstrated that although individual biomarkers had certain diagnostic capabilities (with relatively high AUC values in specific comparisons), their single-marker diagnostic efficiency was limited. This analysis indicated that while individual markers could provide preliminary clues for breast disease status identification, a combined analysis approach was necessary to improve diagnostic accuracy.

### 3.3 Performance of protein-based multimarker panels

**Figure 5:**
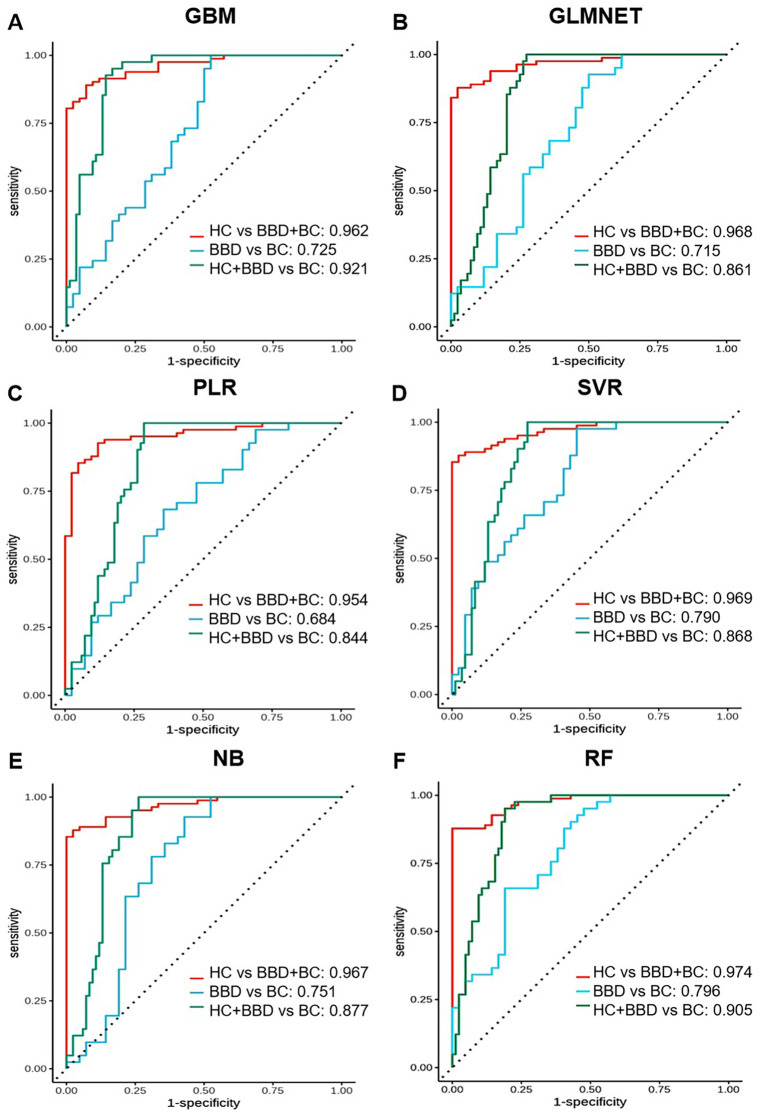
Performance comparison of multi-marker panels based on 10 candidate biomarkers combined with machine learning algorithms. ROC curves are shown for different algorithms in distinguishing different groups (HC vs BBD + BC, BBD vs BC, HC + BBD vs BC). Each sub-figure **(A-F)** corresponds to the performance of one algorithm.

The 180 samples were divided into the training group and the test group at a ratio of 7:3. Based on the 10 candidate biomarkers, we constructed multimarker panels using 6 machine learning algorithms (GBM, GLMNET, PLR, SVR, NB, RF) to optimize the diagnostic performance, as shown in **Figure 5** and **Table 1**. To evaluate the diagnostic performance of models integrating 10 candidate biomarkers with 6 machine learning algorithms (GBM, GLMNET, PLR, SVR, NB, RF), we analyzed three clinical scenarios: distinguishing HC from BBD and BC (HC vs BBD+BC), differentiating BBD from BC (BBD vs BC), and separating the combined non-cancer group (HC+BBD) from BC (HC+BBD vs BC).

**Table 1:** The performance analysis of protein-based multi-marker panels. .

Name	Model type	AUC	Specificity	Sensitivity	Accuracy	Kappa	F1
HC vs BBD+BC	GBM	0.962	1.000	0.886	0.925	0.840	0.939
	GLMNET	0.968	0.944	0.914	0.925	0.836	0.941
	PLR	0.954	0.944	0.914	0.925	0.836	0.941
	SVR	0.969	1.000	0.857	0.906	0.803	0.923
	NB	0.967	1.000	0.857	0.906	0.803	0.923
	RF	0.974	1.000	0.886	0.925	0.840	0.939
BBD vs BC	GBM	0.725	0.941	0.647	0.794	0.588	0.759
	GLMNET	0.715	0.765	0.765	0.765	0.529	0.765
	PLR	0.684	0.824	0.647	0.735	0.471	0.710
	SVR	0.790	0.647	1.000	0.824	0.647	0.850
	NB	0.751	0.647	1.000	0.824	0.647	0.850
	RF	0.796	0.588	0.941	0.765	0.529	0.800
HC+BBD vs BC	GBM	0.921	0.714	1.000	0.808	0.620	0.773
	GLMNET	0.861	0.743	1.000	0.827	0.654	0.791
	PLR	0.844	0.743	1.000	0.827	0.654	0.791
	SVR	0.868	0.800	1.000	0.865	0.723	0.829
	NB	0.877	0.743	1.000	0.827	0.654	0.791
	RF	0.905	0.771	1.000	0.846	0.688	0.810

For the HC vs BBD+BC comparison, all algorithms exhibited strong diagnostic capabilities. The GBM model achieved the highest AUC of 0.962, coupled with perfect specificity (1.000), high sensitivity (0.886), and accuracy (0.925). The GLMNET and PLR models also performed well, with AUC values of 0.968 and 0.954, respectively, and accuracy reaching 0.925. The SVR model showed a high AUC of 0.969 and perfect specificity (1.000), while the NB and RF models had AUCs of 0.967 and 0.974, along with high accuracy and specificity. In the BBD vs BC, which is inherently more challenging, the overall performance was lower compared to HC vs BBD+BC. The NB model stood out with an AUC of 0.751, a sensitivity of 1.000, and an accuracy of 0.824, although its specificity was relatively low at 0.647. The SVR model achieved an AUC of 0.790 and an accuracy of 0.824, while other models like GBM, GLMNET, PLR, and RF had lower AUC values, ranging from 0.684 to 0.725, indicating the difficulty in differentiating between benign and malignant breast conditions. For the HC+BBD vs BC, the models demonstrated reasonable diagnostic performance. The NB model achieved the highest AUC of 0.877, with a sensitivity of 1.000, an accuracy of 0.827, and a specificity of 0.743. The GBM model showed an AUC of 0.921, a sensitivity of 1.000, and an accuracy of 0.808. Other models, such as GLMNET, PLR, SVR, and RF, also had moderate AUC values, ranging from 0.844 to 0.905, suggesting their ability to separate the combined non-cancer group from the cancer group to some extent.

In summary, integrating 10 candidate biomarkers with machine learning algorithms showed promising results, particularly for the HC vs BBD+BC comparison. The GBM and NB algorithms consistently delivered strong performance across different clinical scenarios, with high AUC, accuracy, and balanced sensitivity-specificity. However, the differentiation of BBD from BC remained a challenge, indicating the need for further refinement of the biomarker panel, or enriching the feature dimensions and optimization of the algorithms for this task. These findings support the potential of biomarker-based machine learning models in breast disease diagnosis while emphasizing the importance of tailoring model development to different clinical contexts.

### 3.4 Development of ultrasound radiomics-based models

To further explore the challenges faced by the aforementioned protein marker panels in differentiating BC from BBD, we constructed a machine learning model based on ultrasound radiomics for the BC and BBD populations. In this part, we conducted a series of analyses based on the ultrasound images of 120 patients, including feature selection, feature correlation exploration, and model validation to develop ultrasound radiomics-based models as shown in **Figure 5**.

**Figure 6:**
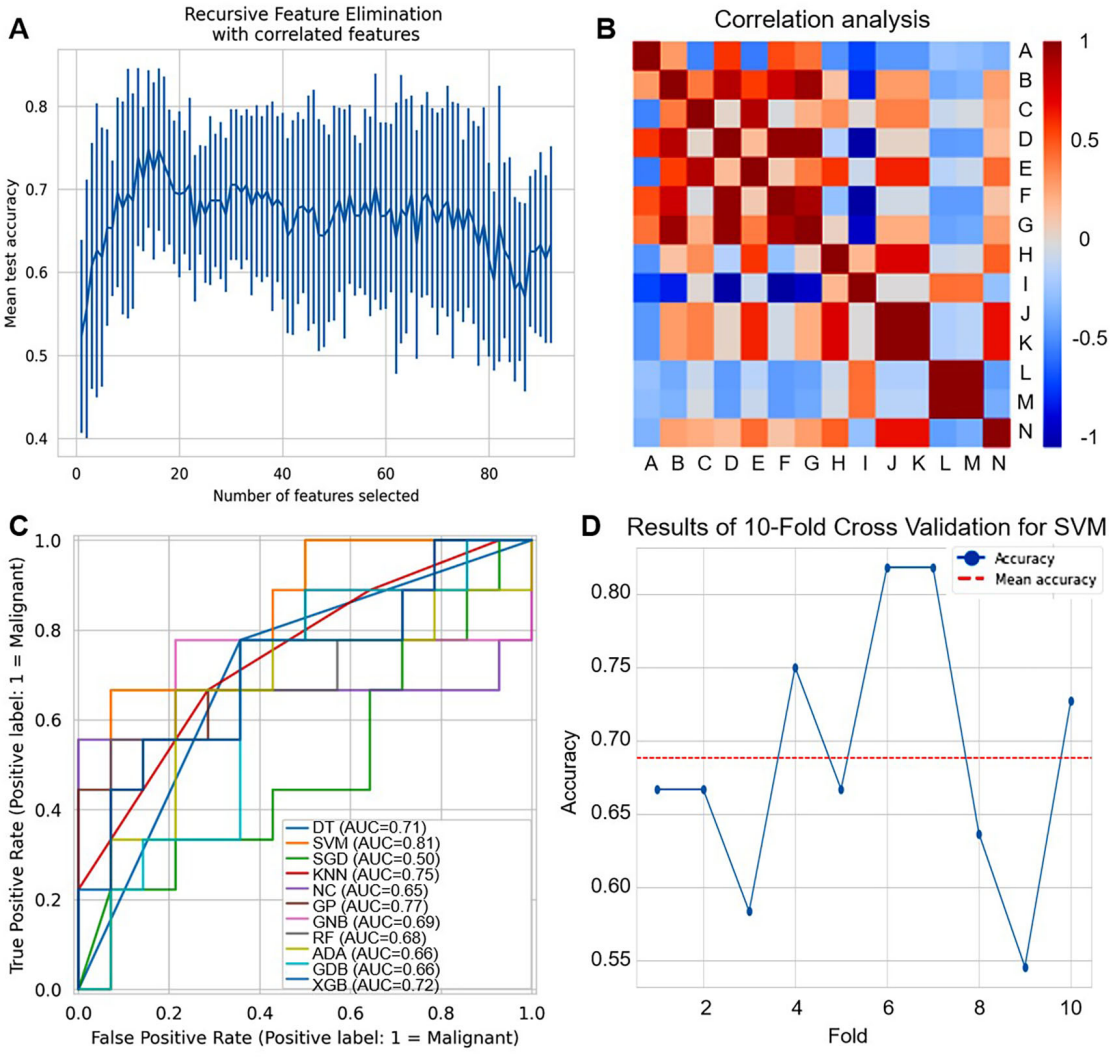
Development of ultrasound radiomics-based models. **(A)** Recursive feature elimination (RFE) plot shows the mean test accuracy as a function of the number of selected radiomic features. **(B)** Correlation heatmap of radiomic features (labeled A-N). Red indicates positive correlation, and blue indicates negative correlation. **(C)** ROC curves compare the performance of different machine learning classifiers for distinguishing malignant (label=1) cases. **(D)** Line plot shows the accuracy of 10-fold cross-validation for SVM across different folds. The red dashed line represents the mean accuracy.

We extracted radiomic features from imaging data. Based on breast ultrasound images and their corresponding annotations, a total of 93 image features were initially retrieved. For feature selection, we employed the RFE method coupled with the Logistic Regression algorithm. RFE results showed that mean test accuracy stabilized at ~0.7-0.8 as the number of radiomic features increased, indicating an optimal subset exists without overfitting, as shown in **Figure 6A**. To ensure robustness, a 10-fold cross-validation strategy was adopted, where samples were randomly shuffled and divided into 10 subsets. Through this process, 14 key features were screened out and retained for subsequent model construction (**Supplementary Table S3**). Correlation analysis revealed strong positive/negative associations among features (labeled A-N), highlighting potential redundancy that could be addressed via selection, as shown in **Figure 6B**.

The ROC curves as shown in **Figure 6C** and **Supplementary Tables S4**, **S5**, showed varying diagnostic capabilities of multiple machine learning classifiers (DT, SVM, SGD, KNN, NC, GPC, GNB, RF, ADA, GDB, XGB). SVM achieved the highest AUC of 0.81, while other classifiers only 0.50-0.77, demonstrating that different classifiers have varying strengths in leveraging the selected radiomic features for breast disease diagnosis. For SVM, as shown in **Figure 6D**, the results of 10-fold cross-validation showed that accuracy fluctuated between 0.55-0.80, with a mean accuracy of around 0.70, reflecting some variability in performance across different data subsets.

### 3.5 Construction and validation of dual-modal diagnostic model

**Figure 7:**
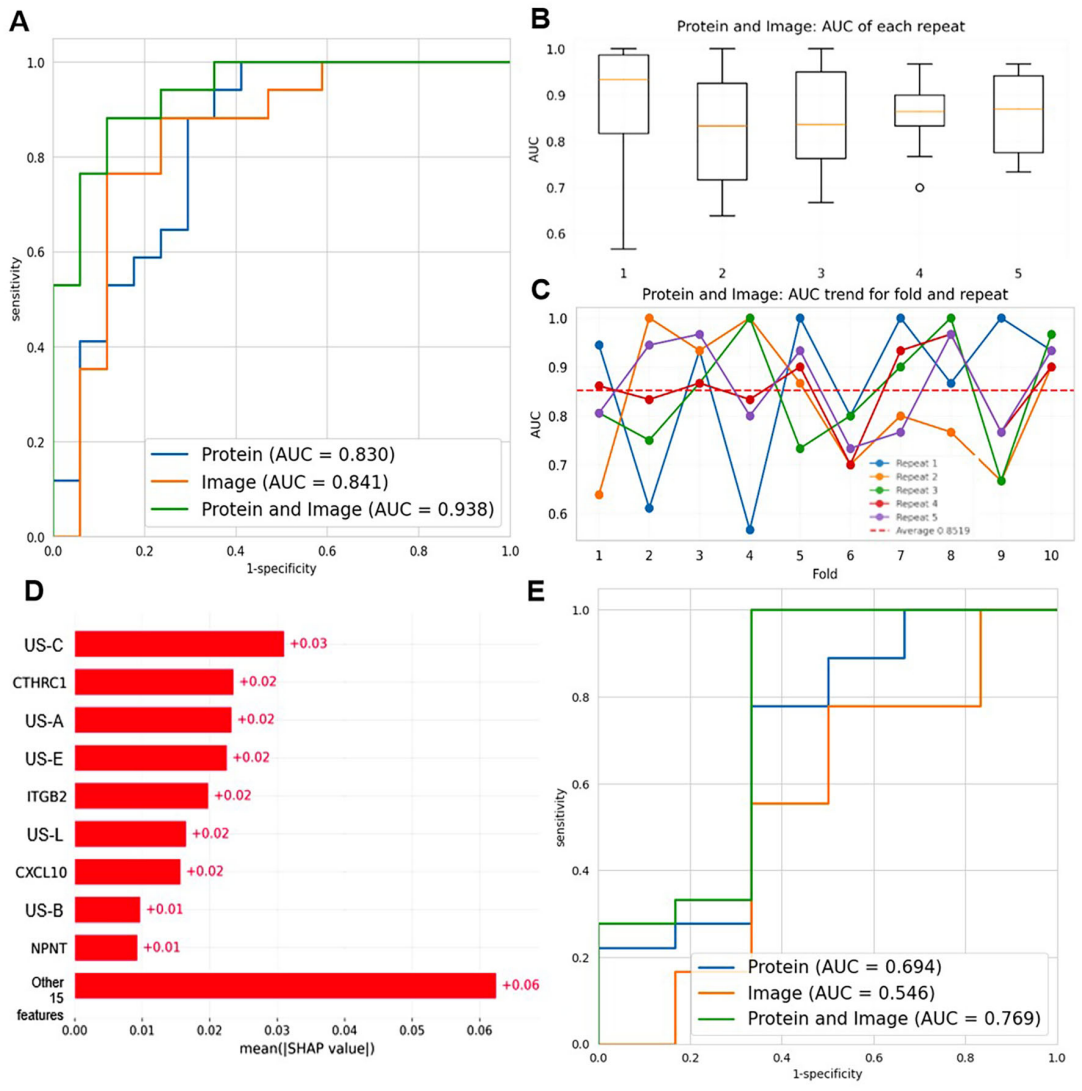
Construction of the dual-modal diagnostic model combining 10 protein biomarkers and 14 radiomic features. **(A)** ROC curves compare the diagnostic performance of protein-only (AUC = 0.830), image-only (AUC = 0.841), and protein+image (AUC = 0.938) models based on SVM. **(B)** Boxplots show the AUC distribution of the protein+image model across 5 repeats. **(C)** The line graph depicts the AUC trend of the protein + image model for 10 folds and 5 repeats. Each color represents a different repeat, and the red dashed line represents the average AUC. **(D)** SHAP summary plot illustrating the mean absolute SHAP values of features in the dual-modal model. **(E)** ROC curves comparing the diagnostic performance of protein-only (AUC = 0.694), image-only (AUC = 0.546), and protein+image (AUC = 0.769) models in a stratified analysis by BI-RADS categories (BI-RADS 3-4A vs 4B-5).

To further improve the diagnostic performance of 10 candidate biomarkers and 14 ultrasound imaging features in the differential diagnosis of BBD and BC patients, we constructed a dual-modal diagnostic model by integrating 10 protein biomarkers and 14 radiomic features based on SVM, as shown in **Figure 7**. The selection of SVM was based on comprehensive performance evaluation across single modalities. In proteomics modeling, SVM showed robust performance in distinguishing BBD from BC (ranking second among tested classifiers), while in radiomics modeling, SVM achieved the highest performance among all evaluated algorithms.

We compared the diagnostic capabilities of three models: protein-only, image-only, and protein+image (dual-mode), as shown in **Figure 7A**. The dual-mode model achieved the highest area under the curve (AUC = 0.938), surpassing the image-only (AUC = 0.841) and protein-only (AUC = 0.830) models. This demonstrated that integrating proteomic and radiomic data significantly enhanced diagnostic accuracy, achieving a better balance between sensitivity and specificity. Then, we assessed the stability of the dual-mode model by 10-fold cross-validation. The AUC values showed relative stability, with a median up to 0.85, indicating consistent performance in multiple runs, as shown in **Figure 7B**. Finally, we analyzed the AUC trend of the dual-mode model across 10 folds and 5 repeats, as shown in **Figure 7C**. Although AUC values fluctuated across folds, they generally remained at a high level (mostly above 0.7). The mean AUC, represented by the dashed red line, was relatively stable, confirming that the dual-mode model maintained good diagnostic performance across different data partitions in cross-validation. In summary, the dual-mode model integrating proteomic and radiomic data outperformed single-modality models in benign and malignant breast disease diagnosis, providing a more effective tool for breast cancer diagnosis.

To enhance the interpretability of the dual-modal model and understand the contribution of individual features, we performed SHAP analysis, as illustrated in **Figure 7D**. The SHAP summary plot revealed the mean absolute SHAP values of features, with US-C, CTHRC1, and US-A being among the top contributors. Positive SHAP values indicated contributions to predicting BC, while negative values favored classification as BBD, validating the biological relevance and discriminative power of key features.

Additionally, we conducted a stratified analysis by BI-RADS categories to explore the model’s performance in specific subgroups (BI-RADS 3-4A vs 4B-5). As shown in **Figure 7E**, the dual-mode model (AUC = 0.769) still outperformed the protein-only (AUC = 0.694) and image-only (AUC = 0.546) models in this stratified setting, though with slightly reduced overall performance compared to the general cohort. This indicated the model’s potential utility across different imaging risk stratifications, even if there is room for improvement in handling more heterogeneous subgroups.

In summary, the dual-mode model integrating proteomic and radiomic data outperformed single-modality models in benign and malignant breast disease diagnosis, provided interpretable insights via SHAP analysis, and showed promise in stratified BI-RADS subgroups, offering a more effective and transparent tool for breast cancer diagnosis.

## 4 Discussion

In this study, we identified 10 novel plasma protein biomarkers via multi-source data integration (including TCGA, CPTAC, and clinical proteomics). Subsequently, we constructed protein-based single-modality models, which showed excellent performance in distinguishing healthy controls from breast disease patients (benign and malignant breast diseases combined) with an AUC of up to 0.974. However, these models showed limited performance in differentiating BBD from BC (AUC 0.684-0.796), prompting the integration of ultrasound radiomics to form a dual-modal model. The dual-modality approach significantly improved benign-malignant discrimination (AUC = 0.938), highlighting a stepwise strategy to address distinct diagnostic needs across clinical scenarios. Our work provides evidence that combining circulating protein markers with ultrasound radiomics can overcome the limitations of conventional diagnostic tools in breast cancer differentiation.

The single-modality models exhibit significant performance differences across various diagnostic scenarios, which stem from the biological characteristics of the markers. Enrichment analysis revealed that the 10-protein panel (CTHRC1, CXCL10, GPATCH4, ITGB2, LMAN2, NPNT, SFRP2, STRBP, TRIM36, VCAN) primarily regulates processes activated early in breast pathogenesis. SFRP2 shows progressive overexpression during the process from healthy individuals to benign individuals and then to cancer individuals. This phenomenon indicates that the dysregulation of the Wnt signaling pathway plays an important role in the development of BC. Studies have shown that SFRP2, as an antagonist of the Wnt signaling pathway, has significantly decreased expression in a variety of cancers (8, 9). In breast cancer, high-frequency methylation of the promoter of the SFRP2 gene leads to its expression silencing. This epigenetic alteration may provide a potential biomarker for the early detection of breast cancer (10). Furthermore, the decreased expression of SFRP2 is associated with the poor prognosis of breast cancer patients, and its tumor suppressor function in breast cancer has been supported (11). CTHRC1 is a soluble protein released by mature osteoclasts, targeting stromal cells to induce osteoblast differentiation (12). In breast cancer, CTHRC1’s involvement in these processes suggests that it could facilitate the structural changes necessary for cancer cells to invade surrounding tissues and form metastatic sites (13). NPNT, VAN, and ITGB2 drive ECM organization, while CXCL10-ITGB2 coordination modulates immune cell recruitment. The biological roles of these 10 biomarkers in the occurrence and development of BC should be further explored in the future. These changes manifest early in breast disease, explaining the panel’s high sensitivity in detecting BBD and BC patients. However, these molecular alterations lack specificity for malignancy, as benign lesions such as fibroadenomas often exhibit similar pathway activation. Consequently, protein biomarkers alone struggled to resolve the BBD vs BC challenge.

Ultrasound radiomics addressed this limitation by capturing malignancy-specific structural consequences (14). Radiomic features such as GLCM entropy, reflecting texture heterogeneity are sensitive to invasive growth patterns absent in benign lesions (15). While radiomics has shown promise in MRI/mammography, ultrasound-based models, which are more cost-effective, have not been promoted (16). In this study, our results showed that the image-only model achieved moderate BBD vs BC discrimination (SVM AUC = 0.81), outperforming the protein panel (AUC = 0.751) by quantifying architectural distortions from stromal desmoplasia or microcalcifications-features directly linked to NPNT-mediated osteoblast differentiation and VCAN-driven ECM stiffening. However, the imaging features of some benign lesions, such as complex hyperplasia, overlap with those of early breast cancer, which affects the diagnostic efficacy of a single imaging modality.

To improve the accuracy of breast cancer diagnosis, researchers are actively exploring the combined application of multimodal markers. Imaging techniques play an important role in the diagnosis of breast cancer. Especially by integrating multiple imaging parameters and molecular biomarkers, the accuracy of diagnosis can be significantly improved (17). Li et al. combined multimodal ultrasound (conventional ultrasound combined with elastography) and tumor marker detection. The results showed that when ultrasound alone diagnosed BC, the AUC was 0.845. When these tumor markers were combined for diagnosis, the AUC increased to 0.928. The AUC of multimodal ultrasound combined with tumor markers for diagnosing BC reached 0.971, significantly improving the accuracy of diagnosing benign and malignant breast lesions (18). Unlike their reliance on less accessible elastography and conventional markers, our model uses widely available conventional ultrasound radiomics plus 10 novel plasma biomarkers, focusing on a stratified workflow (screening via protein model+confirmation via dual-modal model) to balance accuracy and clinical accessibility. In another study, Qiu et al. combined ultrasound imaging with molecular biomarkers to predict the risk of lymph node metastasis in breast cancer patients (19). The results showed that the AUC of the model based on 19 ultrasound features for differentiating non-lymphocytic metastasis from lymph node metastasis was 0.744, and after adding tumor molecular markers, the AUC was 0.793. The AUC, which was significantly higher than that of the clinical risk factor combination, was 0.588. In addition, the dual-modality diagnostic model of ultrasound-biomarkers has also been used to predict the risk of postoperative recurrence and molecular subtype of breast cancer. Song et al. constructed a prediction model for postoperative recurrence risk with an AUC value of 0.8491 (20). The AUC values of TNBC, HER-2, luminal A, and luminal B subtypes were 0.74, 0.92, 0.97, and 0.89, respectively.

In our research, the dual-modal model demonstrated a 10.8% (AUC: 0.938 vs 0.830) performance improvement in the diagnosis of benign and malignant breast diseases compared to the single-modal model, with its diagnostic efficacy far higher than the reported performance of the traditional clinical biomarker CA153 (AUC: 0.6-0.7) (21, 22). Compared to the model in Ishak et al. reported, our dual-modal approach combining protein biomarkers and radiomic features offers a distinct way of multimodal integration (23). While that model focuses on deep learning-based feature extraction from imaging, our approach captures a more comprehensive representation by leveraging both proteomics and radiomics, enhancing diagnostic precision for breast lesions. Regarding deep learning models for breast cancer detection in ultrasound imaging (24), our use of protein biomarkers along with radiomic features provides a more holistic assessment. These models rely solely on ultrasound image patterns, but our approach considers molecular changes in the tumor microenvironment, potentially improving accuracy and providing additional insights. This validates the complementary value of proteomics and imaging data. The combination of ultrasound radiomics and proteomics achieves higher accuracy while reducing costs and intrusiveness, which is a key advantage of scalable screening. Our integration model may created a biological-imaging feedback loop. Proteomics detects early molecular deviations (ideal for screening HC vs BBD+BC). Ultrasound radiomics identifies structural hallmarks of invasion (critical for BBD vs BC). Dual-modality synergistically maps molecular-structural correlations, such as ITGB2-induced ECM degradation is ultimately reflected by irregular ultrasound boundaries. A key strength of this study is its ability to non-invasively capture TME immune dynamics via plasma proteins and ultrasound radiomics. Among the 10 proteins, CXCL10 and TRIM36 stand out as key pro-immune mediators: CXCL10’s upregulation in BC plasma reflects enhanced recruitment of CD8+ T cells and NK cells to the TME, while TRIM36’s upregulation further amplifies anti-tumor immunity by ubiquitinating PD-L1-reducing its surface expression on tumor cells and relieving T cell exhaustion (25, 26). This synergy between immune cell recruitment (CXCL10) and checkpoint inhibition (TRIM36) highlights a coordinated pro-immune signature in BC. In contrast, CTHRC1, VCAN, and NPNT drive immunosuppression: CTHRC1 promotes M2 macrophage polarization to weaken cytotoxic immunity, VCAN blocks T cell penetration via ECM remodeling, and NPNT induces M2 infiltration via αvβ3 integrin (27–29). These three proteins counteract the pro-immune effects of CXCL10 and TRIM36, thereby explaining the TME immune heterogeneity observed in BC. Together, these findings bridge peripheral protein signatures, imaging phenotypes, and TME immunity-offering a tool to infer immunological states of breast lesions without invasive biopsies, which could inform future immunotherapy stratification for BC patients. However, there is currently a lack of direct TME immunoassays to confirm these protein-radiomics-immune links, and we can integrate these data in multi-center collaborations to validate this axis in the future. Additionally, we conducted a stratified analysis by BI-RADS categories. The dual-modal model still outperformed single-modality counterparts in these subgroups, though with slightly reduced AUC compared to the overall cohort. This suggests the model’s utility across different imaging risk stratifications, though further refinement may be needed for more heterogeneous subgroups.

This study still has some limitations. The single-center queue (n=180) may affect the extrapolation of the results and needs to be verified in a multivariate population in the future. In addition, there are observer differences in the manual delineation of ultrasound ROI, and it is necessary to develop an automatic segmentation algorithm based on deep learning. In the future, it is necessary to verify it in multi-center populations and develop automatic segmentation algorithms based on deep learning. Furthermore, the model can be further explored in differentiating benign and malignant conditions and predicting therapeutic effects for different BI-RADS graded populations. The “protein screening to dual-mode diagnosis” approach can be gradually deployed to construct an efficient hierarchical diagnostic system. In addition, the integration of genomic variation and metabolome data is expected to construct a new multi-omics standard for breast cancer molecular typing.

## 5 Conclusion

This study presents a layered diagnostic framework where protein-based models show potential in primary screening, radiomics aids in lesion characterization, and dual-modality integration improves definitive diagnosis. This approach addresses certain limitations of single modalities and offers preliminary insights for precision oncology diagnostics, where different tools could be deployed based on clinical context and diagnostic needs. Further validation with larger sample sizes and external cohorts is warranted to confirm these findings and support broader clinical application.

## Data Availability

The datasets presented in this study can be found in online repositories. The names of the repository/repositories and accession number(s) can be found in the article/**Supplementary Material**.
