# Supplementary material for: A dual-modality machine learning precision diagnostic model integrated radiomics and proteomics for breast cancer
Source: Front Immunol. 2025 Nov 6;16:1665459. doi: 10.3389/fimmu.2025.1665459 (PMC12629940; doi:10.3389/fimmu.2025.1665459)
Supplement: Supplementary file 1 [file Table1.docx]

**Appendices**

**Table S1. The baseline of the 180 individuals enrolled in this study**

| **Variables** | HC (n = 60) | BBD (n = 60) | BC (n = 60) |
| --- | --- | --- | --- |
| **Age, M (Q₁, Q₃)** | 45.00 (33.00,56.00) | 47.00 (42.00,54.25) | 53.50 (46.00,61.25) |
| **First menstrual period age, M (Q₁, Q₃)** | 14.00 (13.00,15.00) | 14.00 (13.00,15.00) | 14.00 (13.00,15.00) |
| **Tumor size, M (Q₁, Q₃)** | - | 1.25 (0.85,1.55) | 13.50 (2.62,19.50) |
| **Node size, M (Q₁, Q₃)** | - | 2.50 (1.00,7.30) | 15.00 (3.40,20.50) |
| **Tumor site, n(%)** |  |  |  |
| Left BC | - | - | 32 (53.33) |
| Right BC | - | - | 28 (46.67) |
| **TNM, n(%)** | - | - |  |
| I | - | - | 37 (61.67) |
| II | - | - | 14 (23.33) |
| III | - | - | 8 (13.33) |
| IV | - | - | 1 (1.67) |
| **Molecular subtyping, n(%)** | - | - |  |
| HER-2+ | - | - | 16 (26.67) |
| Luminal A | - | - | 7 (11.67) |
| Luminal B | - | - | 37 (61.67) |
| **Histological type, n(%)** | - |  |  |
| Intraductal papilloma | - | 10 (16.67) |  |
| Adenosis of breast | - | 9 (15.00) | - |
| Cyclomastopathy | - | 16 (26.67) | - |
| Fibroadenoma | - | 18 (30.00) | - |
| Invasive ductal carcinoma | - | - | 52 (86.67) |
| Invasive solid papillary carcinoma | - | - | 3 (5.00) |
| Invasive lobular carcinoma | - | - | 2 (3.33) |
| Invasive mucinous carcinoma | - | - | 1 (1.67) |
| Metastatic carcinoma of lymph nodes | - | - | 2 (3.33) |
| **Grade, n(%)** | - |  |  |
| 1 | - | - | 8 (13.33) |
| 1-3 | - | - | 1 (1.67) |
| 2 | - | - | 30 (50.00) |
| 3 | - | - | 10 (16.67) |
| Unknow | - | - | 11 (18.33) |
| **BI-RADS, n(%)** | - |  |  |
| 2 | - | 1 (1.67) | 0 (0.00) |
| 3 | - | 19 (31.67) | 4 (6.67) |
| 4A | - | 20 (33.33) | 7 (11.67) |
| 4B | - | 9 (15.00) | 22 (36.67) |
| 4C | - | 2 (3.33) | 23 (38.33) |
| 5 | - | 0 (0.00) | 4 (6.67) |

**Table S2. Plasma Detectability Validation of Candidate Protein Biomarkers**

| **Protein** | **Subcellular Location** | **Source** |
| --- | --- | --- |
| CTHRC1 | Secreted | UniProt |
| CXCL10 | Secreted | UniProt |
| GPATCH4 | Predicted-outside | TMHMM |
| ITGB2 | Blood-MS detected | THPA |
| LMAN2 | Blood-MS detected | THPA |
| NPNT | Secreted | UniProt |
| SFRP2 | Secreted | UniProt |
| STRBP | Predicted-outside | TMHMM |
| TRIM36 | Predicted-outside | TMHMM |
| VCAN | Secreted | UniProt |

**Table S3. Corresponding table of label and features**

| **Label** | **Feature** |
| --- | --- |
| A | original_firstorder_10Percentile |
| B | original_firstorder_90Percentile |
| C | original_firstorder_InterquartileRange |
| D | original_firstorder_Mean |
| E | original_firstorder_MeanAbsoluteDeviation |
| F | original_firstorder_Median |
| G | original_firstorder_RootMeanSquared |
| H | original_glcm_ClusterShade |
| I | original_gldm_LargeDependenceLowGrayLevelEmphasis |
| J | original_glrlm_GrayLevelVariance |
| K | original_glszm_GrayLevelVariance |
| L | original_glszm_LargeAreaEmphasis |
| M | original_glszm_ZoneVariance |
| N | original_ngtdm_Complexity |

**Table S4. Results of 10-Fold Cross Validation for ultrasound-based multiple machine learning classifier**

| **Classifier** | **mean_accuracy** | **std_accuracy** |
| --- | --- | --- |
| DT | 0.567 | 0.151 |
| SVM | 0.688 | 0.086 |
| SGD | 0.601 | 0.122 |
| KNN | 0.668 | 0.070 |
| NC | 0.688 | 0.115 |
| GP | 0.660 | 0.076 |
| GNB | 0.653 | 0.047 |
| RFC | 0.654 | 0.116 |
| ABC | 0.642 | 0.111 |
| GBC | 0.646 | 0.125 |
| XGB | 0.662 | 0.158 |

**Table S5. Varying diagnostic capabilities of ultrasound-based multiple machine learning classifier**

| **Model_name** | **Accuracy** | **Sensitivity** | **Specificity** | **AUC_ROC** |
| --- | --- | --- | --- | --- |
| DT | 0.696 | 0.778 | 0.643 | 0.710 |
| SVM | 0.826 | 0.667 | 0.929 | 0.810 |
| SGD | 0.652 | 0.222 | 0.929 | 0.500 |
| KNN | 0.696 | 0.667 | 0.714 | 0.746 |
| NC | 0.826 | 0.556 | 1.000 | 0.651 |
| GP | 0.783 | 0.444 | 1.000 | 0.770 |
| GNB | 0.783 | 0.778 | 0.786 | 0.690 |
| RF | 0.783 | 0.556 | 0.929 | 0.683 |
| ABC | 0.739 | 0.667 | 0.786 | 0.659 |
| GBC | 0.696 | 0.778 | 0.643 | 0.659 |
| XGB | 0.696 | 0.778 | 0.643 | 0.722 |
